# Vascular complications with injection implants: treatment with hyperbaric chamber

**DOI:** 10.1080/23320885.2021.1933492

**Published:** 2021-07-01

**Authors:** Danuza Dias Alves, Honório Sampaio Menezes, Roberto Chacur, Rodrigo Cadore Mafaldo, Nívea Maria Bordin da Silva Chacur, Leandro Dias Gomes, Raíssa Nardi, Gabriella Andressa Marchesin de Castro, Vanessa Pletsch Brendler Borges, Manuela Guimarães Dias Gomes

**Affiliations:** a Clínica Leger, Rio Grande do Sul, Brazil; b Clínica Leger, Rio de Janeiro, Brazil; c Instituto de Oxigenoterapia Hiperbárica do Brasil, Porto Alegre, Brasil

**Keywords:** Necrosis, treatment in a hyperbaric chamber, vascular occlusion, facial filler

## Abstract

**Introduction:**

The use of facial fillers has increased over the years because they are less invasive and present lower risks and faster results, along with shorter recovery time.

**Objective:**

This study aimed to analyze the use of hyperbaric oxygen therapy as a possible treatment for vascular complications resulting from facial fillers.

**Methodology:**

This is a retrospective study of a series of patients treated at with hyperbaric oxygen therapy at the Brazilian Institute for Hyperbaric Oxygen Therapy (Instituto de Oxigenoterapia Hiperbárica do Brasil), after vascular complications from facial fillers.

**Conclusion:**

The association of oxygen therapy in a hyperbaric chamber with the conventional protocol for treating vascular occlusion from facial filler procedures was found to be effective.

## Introduction

The use of facial fillers has increased over the years, as they are non-invasive. However, one of the most feared complications is necrosis due to vascular obstruction. Impending necrosis can be associated with several soft tissue fillers, happening after approximately 0.001% of the procedures. Therefore, extensive knowledge of the vascular anatomy of the face is essential, especially of areas with terminal vessels [1,2].

Injection complications are rare; nevertheless, when there is damage to or compression of vessels, or even intravascular obstruction by the injected material, they occur. There are several medical recommendations on how to treat imminent injection-induced necrosis which include: immediate discontinuation of the injection and treatment with hyaluronidase; application of hot compresses; local wound treatment; and use of nitroglycerin, non-steroidal anti-inflammatory drugs (NSAIDs), anticoagulants, or antibiotics; among others. In severe cases of skin necrosis, hyperbaric oxygen therapy (HBOT) should also be considered [1,2].

Hyperbaric oxygen therapy is regulated in Brazil by the Federal Council of Medicine (CFM) since 1995 (CFM No. 1,457/1995), following safety, quality, and ethics guidelines developed and reviewed by the Brazilian Society of Hyperbaric Medicine. It is a therapeutic means for delivering oxygen to the lungs at a pressure higher than the standard atmospheric pressure above sea level. This treatment can be administered in mono- (for a single patient) or multi-place (for multiple patients) chambers [3–6].

Administration of oxygen under pressure leads to very high systemic oxygen concentrations. Soft-tissue penetration of some antibiotics is improved in hyperbaric conditions. Moreover, HBOT's vasoconstrictor properties can lead to reduced edema and improved tissue viability in acute wounds, such as compromised skin grafts [5,7].

Hyperbaric oxygen therapy consists of placing the patient in a pressurized chamber which may range from 2 to 3 atmospheres of pressure (atm), that is, up to three times greater than that at sea level. High pressure combined with the exposure to 100% oxygen has many physiological effects on the body. Several of them have been shown to improve the healing of complicated chronic wounds [7].

One of the most dramatic effects is a higher concentration of oxygen in the plasma. The normal oxygen concentration in plasma at sea level is 3 ml/L, while at 3 atm, with 100% oxygen, it may reach approximately 60 ml/L. This increase also results in the release of higher concentrations of oxygen in the ischemic tissue. The only absolute contraindication for this treatment is an untreated pneumothorax [7].

This work aimed to qualitatively evaluate the evolution of patients submitted to a hyperbaric oxygen therapy for the treatment of vascular occlusions resulting from the use of soft-tissue fillers.

## Methodology

This is a retrospective study of a series of patients treated at Clínica Leger who underwent treatment with hyperbaric oxygen therapy at the Brazilian Institute for Hyperbaric Oxygen Therapy (Instituto de Oxigenoterapia Hiperbárica do Brasil) after vascular complications from facial fillers. Descriptive data of each patient, which included their clinical history, data from the event, and treatment carried out, were collected. This study was approved by the Veiga de Almeida University Research Ethics Committee under protocol number 28930620.0.0000.5291. There was no need for patients to sign the informed consent form, the data were taken from clinical doctors.

### Clinical case 1

A female, 47-year-old patient underwent treatment for nasolabial folds. Polymethylmethacrylate (PMMA) 10% was injected with a 22 G microcannula. Local anesthesia of lidocaine 2% was administered, then the cannula was introduced through the right oral commissure. At that moment, the patient moved abruptly and reported feeling pain. Although the cannula had already been inserted, the filler had not yet been injected. A retrograde injection of 0.4 mL of PMMA in total was administered. As soon as injected, when removing the cannula, the responsible doctor noticed edema in the region of the nasolabial folds, which spread to the malar region, and the patient reported discomfort at the site. Then, the immediate formation of a small hematoma was observed in the cannula path and, after five minutes, the skin presented a livedoid aspect in the entire cheek region. The protocol for treating occlusion, which consists of severe massage in the region, application of hot compresses, prescription of acetylsalicylic acid (ASA) 300 mg, prednisone 20 mg and prophylactic antibiotic, was started at this moment. The doctor massaged vigorously the region and applied hot compresses. After action was taken, the patient remained in the clinic under observation for approximately one hour. As the patient reported that the pain had ceased, and a relative improvement was observed; she was prescribed antibiotics and sent home.

**Figure 1.**
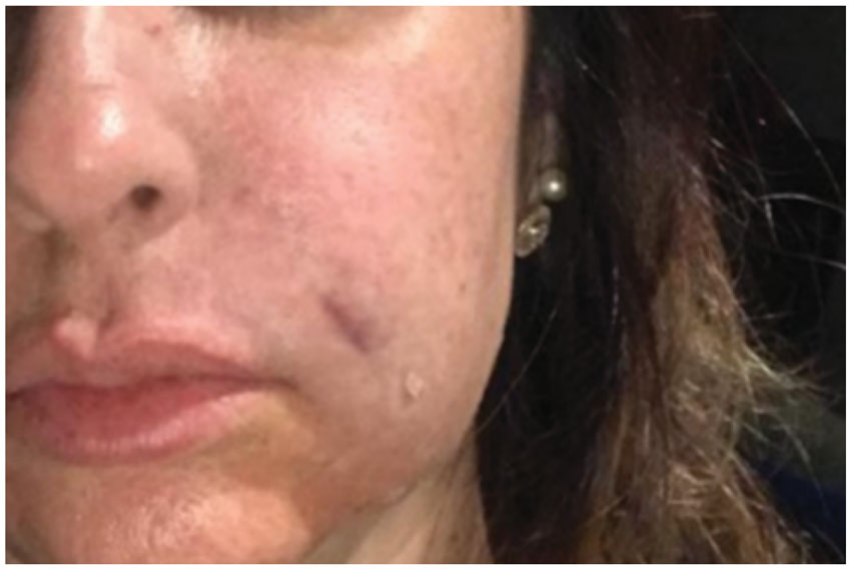
24 September 2018. Immediate post-procedure. Edema in the nasolabial folds with hematoma along the cannula. Skin presented a livedoid aspect.

**Figure 2.**
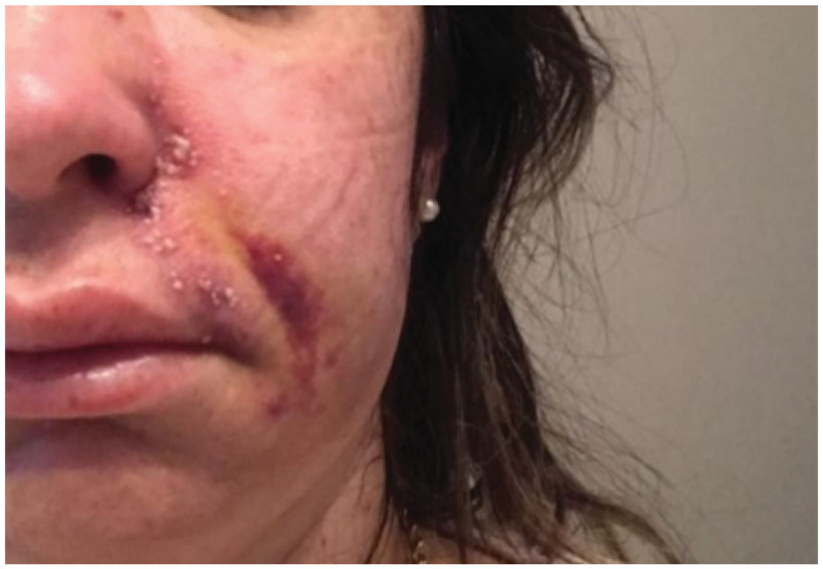
27 September 2018. Third day after procedure. Lesions evolved to pustules in the nasal ala.

**Figure 3.**
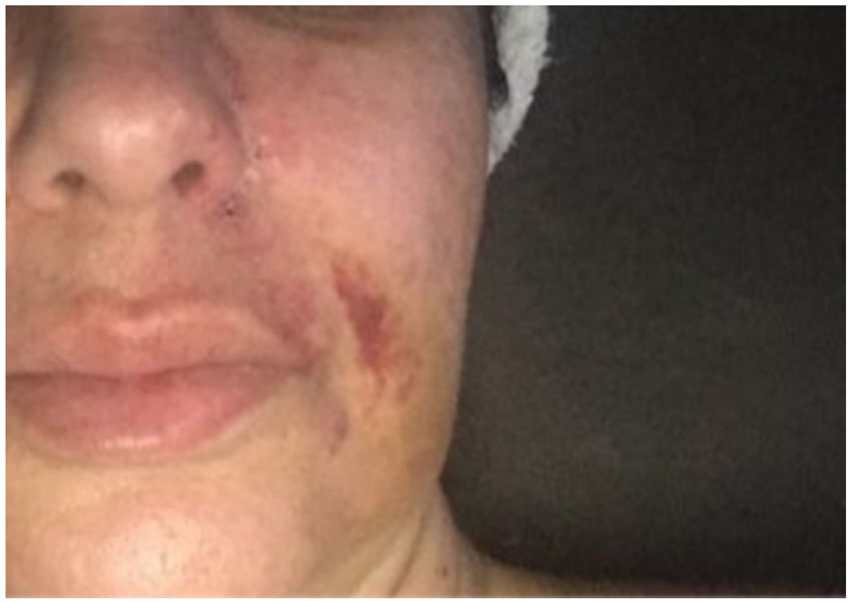
29 September2018. Result after the 3rd hyperbaric chamber session.

**Figure 4.**
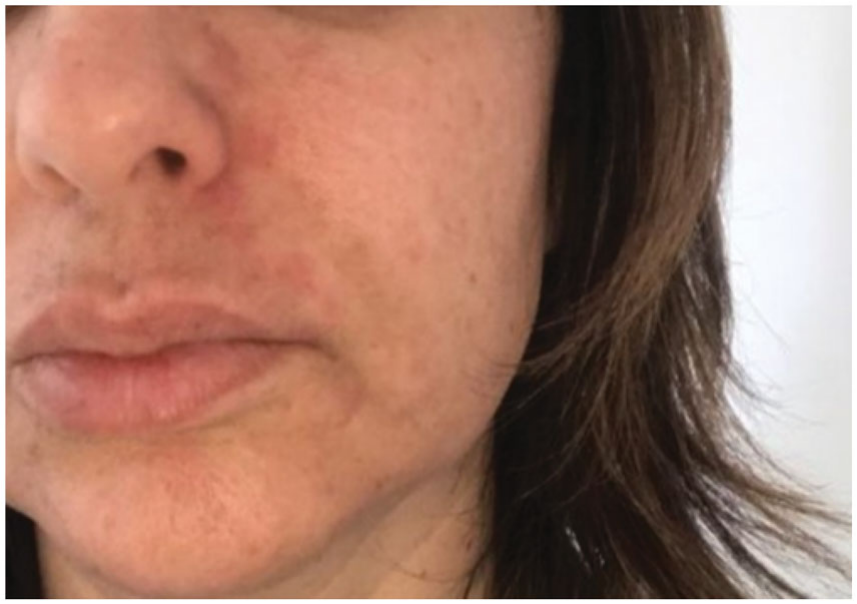
6 October 2018. Result after the 7th session in hyperbaric chamber. Patient was discharged from treatment because full reperfusion of the affected area had been achieved.

**Figure 5.**
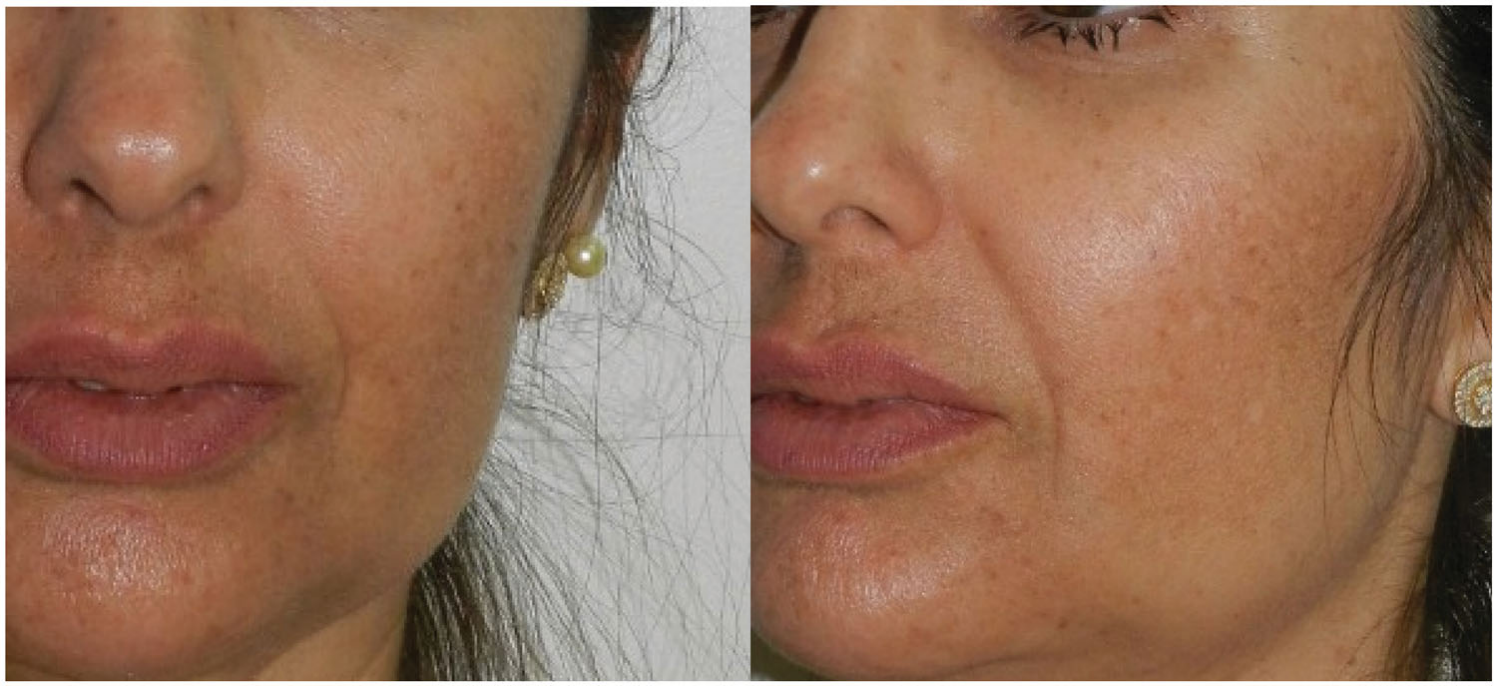
Follow-up one month after the procedure.

On the following day, the doctor asked the patient for a photo of the affected region; it looked better, but there was still quite a significant edema. On the second day after the procedure, there was an improvement of the edema, and the patient reported being happy with the evolution of the case. On the third day following the procedure, the patient progressed to develop pustules in the nasal ala, which is compatible with tissue distress and epidermolysis. The patient was then immediately seen by the doctor, who continued with the protocol and prescribed acetylsalicylic acid (ASA) 300 mg and prednisone 20 mg daily. On clinical examination, ischemic right buccal mucosa and hyperemic skin were observed from the right upper lip, nasolabial fold, nasal ala, up to the glabella; delimiting the region of the angular branch of the facial artery. In addition to the traditional protocol for the treatment of arterial occlusion, the patient started oxygen therapy in a hyperbaric chamber, accompanied by the doctor responsible for the filling procedure and the one in charge of the oxygen therapy. The patient started the treatment on the third day after the procedure, being subjected to a pressure of 2.4 atm in a multi-place chamber with 100% oxygen inhalation for 90 min, and reported relative improvement. The treatment was carried out daily for another six days, following the recommendation of the doctor responsible for the oxygen therapy. In the daily monitoring of the patient by the doctor, a significant improvement between each session of the hyperbaric chamber could be seen. In the fifth session (on the eighth day after the procedure), the doctors responsible for the injection and the oxygen therapy observed that the patient's skin had already recovered approximately 100%, with no signs of scarring. The patient underwent two more sessions and then the doctor at the oxygen therapy service considered the treatment to be successfully completed, as it had reversed 100% of the arterial occlusion with total restoration of the affected skin and tissues (Figures 1–5).

### Clinical case 2

A female, 39-year-old patient had a prosthesis in her chin, which was insufficient to satisfy her desire. In the medical evaluation, filling with PMMA was indicated to fill the region. After the consent form was signed by the patient, the procedure was performed. Local anesthesia with lidocaine 2% was administered in the mental region, and a 22 G microcannula was inserted through the entry point to inject PMMA 10%. The correction of a depression caused by a prosthesis on the left side of the chin started, however, when 0.3 mL of PMMA 10% was injected, a hematoma and cyanosis were observed on the chin. The patient remained under observation for approximately five minutes by the responsible doctor. Hyperemia extending from the side of the chin, in the mandibular region, to the nasal ala on the left side was observed, which is very characteristic of the occlusion of the facial artery. Thereby, the doctor started the occlusion protocol, massaging the site and applying hot compresses.

At no time the patient reported feeling pain. She remained under observation for another hour at the clinic. The doctor, in suspecting arterial occlusion, followed the treatment protocol and prescribed antibiotics, corticosteroids 20 mg, and acetylsalicylic acid (ASA) 300 mg per day before discharging the patient. Twelve hours after the procedure, the doctor requested the patient to send a photo of the affected area. The patient had more intense hyperemia in the region of the facial artery and a significant hematoma in the chin, which led the doctor to the diagnosis of vascular occlusion. Therefore, the doctor referred and accompanied the patient to a hyperbaric oxygen therapy center. The treatment, overseen by the doctor responsible for the oxygen therapy, was carried out to reverse the region's arterial suffering. The patient was subjected to 2.4 atm of pressure in a multi-place chamber with 100% oxygen inhalation for 90 min. On the third day of treatment, despite the external part of the mucosa appearing cyanotic, there was a significant reduction in both the hematoma and the hyperemia in the region. On the fifth day of the therapy, that is, the sixth following the procedure, the patient presented recovery of 100% of the blood flow rate, as monitored by the doctors responsible for the procedure and the oxygen therapy treatment.

**Figure 6.**
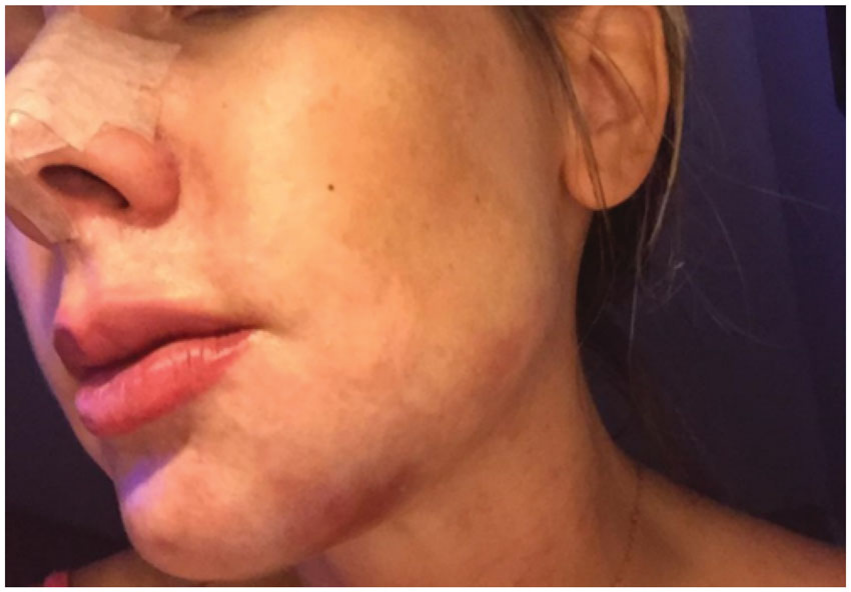
9 January 2019. Immediate post-procedure. Hematoma and skin with cyanotic/hyperemic aspect.

**Figure 7.**
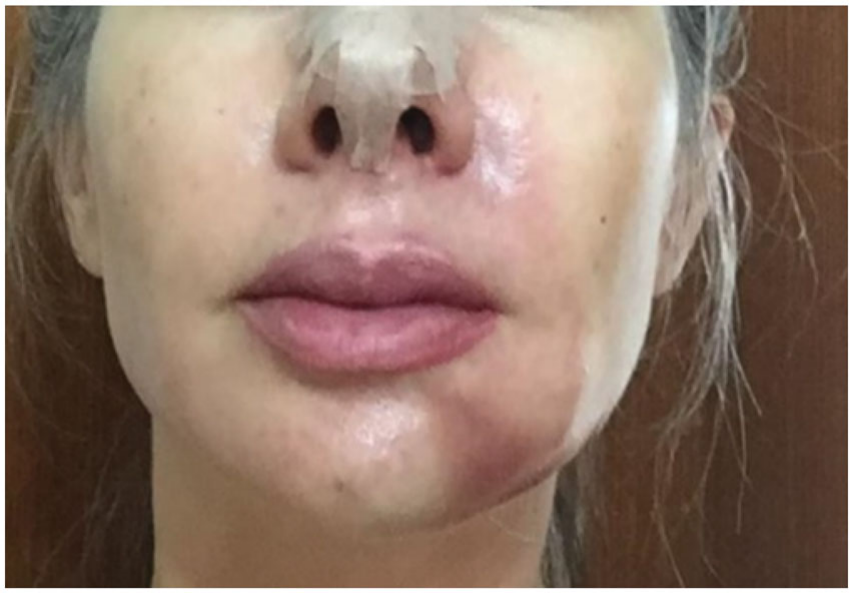
10 January 2019. Twelve hours after the procedure. Severe hyperemia in the vascular artery region and significant chin hematoma.

**Figure 8.**
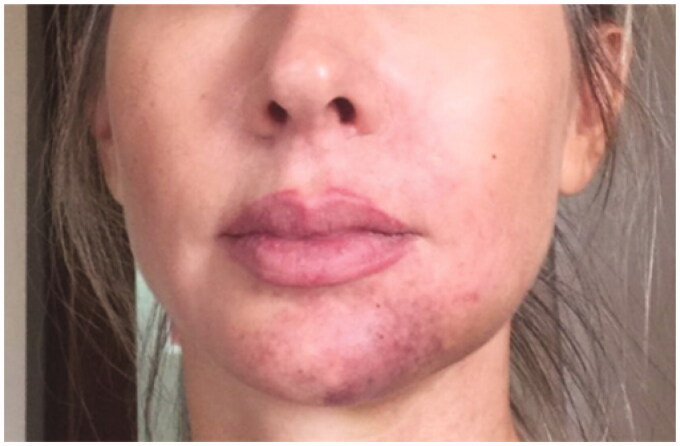
13 January 2019. Result after the 3rd session in the hyperbaric chamber.

**Figure 9.**
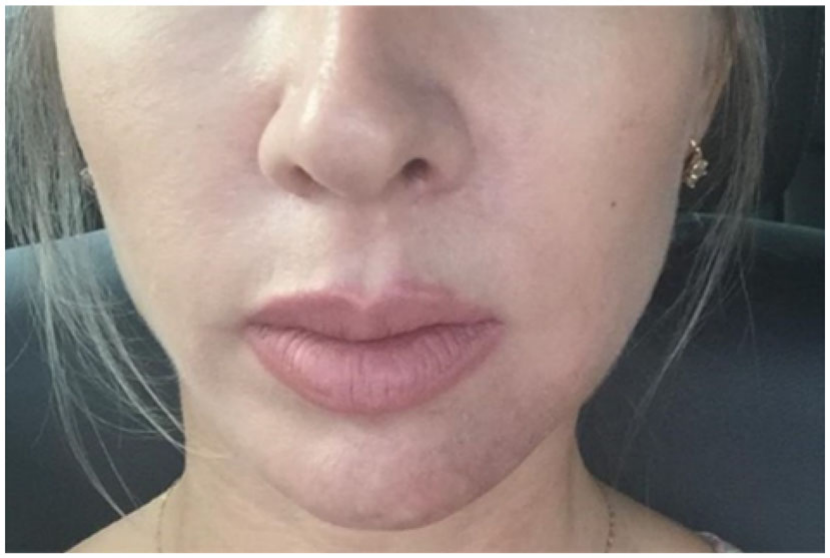
5 January 2019. Result after the 5th session in the hyperbaric chamber. Patient was discharged from treatment because full reperfusion of the affected area had been achieved.

**Figure 10.**
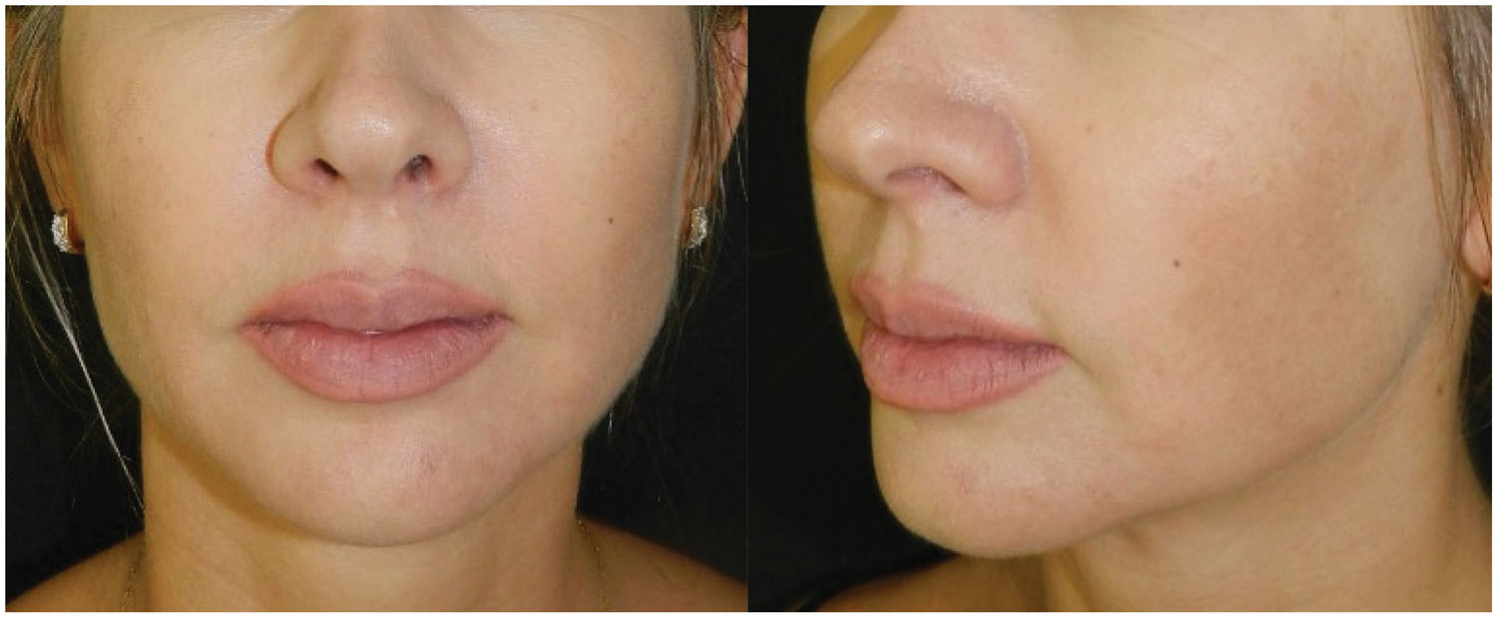
Follow-up one month after the procedure.

After the fifth session of oxygen therapy, the patient was discharged. Seven days after the procedure, she contacted the doctor once more to report wellness during a car trip and 100% of the region healed, without any visible scarring (Figures 6–10).

## Discussion

The physiological and therapeutic effects of hyperbaric oxygen therapy are related to its method of use, which consists of an environment with higher atmospheric pressure and administration of pure oxygen (100%) to provide an ideal setting for wound healing [8]. Based on scientific works and clinical data of more than 10 years, this study analyzed the use of oxygen therapy in a hyperbaric chamber to treat vascular occlusions resulting from filler procedures.

According to a cross-sectional study conducted at a hyperbaric center in the city of Salvador (Bahia, Brazil) in which 200 medical records of patients treated with HBOT were analyzed, patients’ health conditions influence the result of oxygen therapy treatments. Diabetes, for instance, is a significant factor which can affect wound healing due to structural changes in the cell membrane [8]. Therefore, this study considered healthy patients who did not have any pathology that could negatively affect the outcome of treatments with HBOT.

Leach et al. demonstrated that the hyperbaric chamber, when used at a pressure equal to or lower than 300 kPa for approximately 120 min, is safe. Severe symptoms of nervous system were seen in 1–2% of the treated patients. The most common side effect, seen in approximately 20% of the cases, was reversible myopia. It occurs due to oxygen toxicity and can last for weeks or months [9]. Thus, the use of oxygen therapy associated with the standard protocol for reversing vascular occlusion is safe when used properly and prescribed by a qualified medical professional.

A study by the International Society of Aesthetic Plastic Surgery (ISAPS) from 2018 showed a 10.4% increase in non-invasive procedures, such as filler injections. With a higher demand for these procedures, the rate of complications also increased [10]. In general, complications related to soft-tissue filler procedures are associated with unqualified professionals; however, even when carried out by experienced professionals, they can cause reactions which range from small and self-limited to severe. These require immediate medical management, which may include hyperbaric oxygen therapy [11]. Hence, considering the studies found in the literature and the more than 10 years of experience at the clinic, it is possible to validate the use of HBOT for treating complications of vascular occlusion from filler procedures.

## Conclusion

This study showed the reversal of vascular occlusion resulting from facial filler procedures in patients treated with hyperbaric oxygen therapy. HBOT has proven to be effective in oxygenating ischemic tissues, reducing edema, and healing lesions, thereby, restoring the affected skin and tissues without leaving visible scars. It is essential that the treatment is prescribed by a qualified doctor and carried out under the responsibility of a professional specialist in oxygen therapy, who must be experienced in handling the chamber, thus, ensuring the safety of the patient, as well as reducing adverse effects associated with HBOT.

The positive clinical effects of oxygen therapy in hyperbaric chambers in the treatment of facial vascular complications were evident and its use seems very promising for future treatment protocols for vascular occlusion.
